# Polymers Sorption Properties towards Photosynthetic Pigments and Fungicides

**DOI:** 10.3390/ma14081874

**Published:** 2021-04-09

**Authors:** Małgorzata Tatarczak-Michalewska, Jolanta Flieger, Justyna Kawka, Wojciech Płaziński, Tomasz Klepka, Piotr Flieger, Monika Szymańska-Chargot

**Affiliations:** 1 Department of Analytical Chemistry, Medical University of Lublin, Chodźki 4A, 20-093 Lublin, Poland; justyna.kawka@umlub.pl; 2 Jerzy Haber Institute of Catalysis and Surface Chemistry, Polish Academy of Sciences, Niezapominajek 8, 30-239 Krakow, Poland; wojtek_plazinski@o2.pl; 3 Department of Technology and Polymer Processing, Faculty of Mechanical Engineering, Lublin University of Technology, Nadbystrzycka 36, 20-618 Lublin, Poland; t.klepka@pollub.pl; 4 Interfaculty Centre for Didactics, Medical University of Lublin, Jaczewskiego 4, 20-090 Lublin, Poland; piotr.flieger@umlub.pl; 5 Institute of Agrophysics, Polish Academy of Sciences, Doświadczalna 4, 20-290 Lublin, Poland; m.szymanska@ipan.lublin.pl

**Keywords:** chlorophylls, fungicides, solid–liquid extraction, polymers, sustainable chemistry

## Abstract

In the present work, extraction with a solvent (cold acetone) was used to extract the assimilation pigments from spinach leaves. Then, the sorption capacity of selected plastics granules (polyvinyl chloride—PVC, polypropylene—PP, polyethylene—PE of different densities) was tested for the selective isolation of chlorophylls. Quantification of chlorophylls by HPLC (Zorbax Eclipse XDB-C18 column, the mobile phase: Acetonitrile/methanol/ethyl acetate 6:2:2, *v*/*v*) was based on chlorophyll-a content as the most common chlorophyll. The performed experiments prove that PVC containing electronegative chlorine exhibits favorable interactions toward chlorophyll by creating stable molecular complexes. The Fourier Transform Raman Spectroscopy (FT-Raman) and the molecular modeling were used to elucidate the structure of the created complexes. The optimal extraction requirements, the mass of sorbent, water-acetone ratio, time, and the composition of the elution solvent were all established. The optimized extraction conditions ensured a maximum extraction yield of chlorophylls of 98%. The chlorophyll-rich sorbent was re-extracted by acetone, leading to the recovery of 91% of chlorophylls in one step, adding the possibility of its re-use. The proposed effective and ecological method of obtaining the green dye from plants is cheap, simple, and efficient, avoiding organic solvents, utilizing the most widely used synthetic polymers in the world, being products difficult for utilization. The possibility to remove chosen fungicides cyprodinil, chlorothalonil, and thiabendazone from plant extract by PVC was also examined. The described method proposes a new application of synthetic polymers, which meets the criteria of sustainable green chemistry, simultaneously reaching the growing demand for pure natural compounds in the pharmaceutical and food industries.

## 1. Introduction

Chlorophylls are organic chemicals found in the cells of plants, algae, and photosynthetic bacteria that are responsible for the characteristic green color [1]. In a chlorophyll molecule, four pyrrole rings (I to IV) connected by methine bridges (-CH=) are bound into a large, flat, symmetrical molecule with a single magnesium ion, which is coordinated with the four pyrrole rings. Ring IV is esterified with a phytol group which renders the molecule hydrophobic. Chlorophyll *a* in the 3 position has a methyl group, but taller plants and algae use an additional form of chlorophyll, chlorophyll *b*, which has a formyl group in place of the methyl group. Chlorophyll *a* is the main pigment involved in the photosynthetic process in higher plants and algae. Two chemical structures of chlorophyll are present in plants: Chlorophyll *a* and chlorophyll *b*, usually in a ratio of 3:1.1.

Due to the presence of double bonds in the molecule, chlorophylls are effective photoreceptors with a characteristic high absorption of visible light in two regions around 400 and 600 nm [2]. A Chlorophyll *a* was absorbed at approximately 420–440 nm (Soret band) and approximately 650–670 nm (*Q* band) [3]. The number of naturally occurring chlorophylls may still not be fully known. The main elements of photosynthesis involved in photosynthesis are chlorophyll *a* and chlorophyll *b*, but we also know other types of chlorophyll pigments, such as chlorophyll *c*, chlorophyll *d*, chlorophyll *f*, protochlorophyll, bacteriophyll, and chlorobium chlorophyll [4].

Chlorophylls exhibit strong biological antioxidant, antibacterial, and anti-inflammatory properties. Their stimulating and regenerating effect on tissue growth has also been demonstrated. The effectiveness of photodynamic therapy with the use of chlorophyll *a* in oncology and dermatology, confirms a wide range of applications also in the field of medical sciences [5,6,7,8]. Chlorophylls isolated from plants are also commonly used as safety dyes in the food industry [9].

It is possible to extract dyes from natural raw materials, i.e., green plants, algae, bacteria, cyanobacteria in the extraction process with an appropriate solvent and properly selected method, e.g., in the Soxhlet apparatus, liquid extraction using ultrasound assisted extraction (UAE), microwave assisted extraction (MAE), or supercritical fluid extraction (SFE).

For large-scale extraction of natural chlorophylls from plants, acetone, dimethylsulfoxide, dioxane, and dimethylformamide are usually used as preferred solvents. Most pigments can be removed in the process, which is called as defatting. Extraction using solvents like petroleum benzene, n-hexane, dichloromethane, petroleum ether, n-hexane, carbon tetrachloride removes most of the lipophilic compounds like chlorophylls together with fatty materials. After a drying step, biomass is extracted with methanol–petroleum ether mixtures at the first step, then finally with acetone. Most of the solvents which are used in industry are volatile and toxic, leading to environmental risks. Furthermore, such extraction procedures are multi-step, and thus costly, and are of low selectivity, resulting in low purity levels and yields [10,11].

The coloring matter of plant extract can be removed by adding a sufficient quantity of activated charcoal or activated bentonites [12]. However, the use of activated bentonites and charcoal agents has the drawback of non-specific adsorption that results in losses of desired bioactive compounds. In the case of organic solvents such as hexane or carbon tetrachloride, which are typically used for separating chlorophylls, it should be emphasized that they are toxic to humans and to the environment and have low efficacy in chlorophyll removal, and require multistep extraction procedures.

Purification of plant extracts from pigments can be accomplished by several techniques such as fractionation, saponification [13], and chromatography [14,15,16]. Although these procedures are rather simple, stress conditions such as temperature or pH may cause chlorophyll degradation [13].

To remove the green pigment at the micro-scale, SPE cartridges containing graphitized carbon black (GCB) and primary secondary amine (PSA) can be applied. However, this is a rather expensive clean-up method that is used in QuEChERS sample preparation. This method is used in laboratory to remove chlorophylls because they often interfere with the analysis of bioactive extract components or in pesticide residue analysis [17,18], strongly influencing the background ion current in mass spectrometry (MS) detectors. That is why additional extensive clean-up step(s) are required before analysis, for improvement of the results accuracy.

In 2013, Batlokwa et al. [19] elaborated on a novel molecularly imprinted polymer (MIP) for the selective removal of chlorophyll from heavily pigmented green plant extracts. The initial chlorophyll absorbance of the methanolic, extract was reduced by 98% after the addition of the of 850 mg MIP in the 5 mL of 10% (*w*/*v*) chlorophyll standard solutions. More recently, a liquid–liquid extraction system composed of ethanol, hexane, and water, which was tested in terms of their ability to selectively separate chlorophylls and xanthophylls to opposite phases in a single step [20]. Phaisan et al. [21] proposed an oil-based system for removing chlorophyll from the extract of *C. odorata*. This requires simple processing based on the partitioning technique. According to the authors statement, palm oil showed an efficiency higher than hexane for chlorophyll removal and high recoveries of the beneficial phytochemicals. More recently, Leite et al. [22] showed the usefulness of aqueous solutions of non-ionic surfactants with hydrophilic-lipophilic balance (HLB) ranging between 10 and 13, to extract chlorophylls from biomass.

Unfortunately, pesticides widely used for protecting crops against fungal diseases during storage and transport are able to contaminate the plant extracts prepared by the use of solvents such as ethyl acetate, aqueous acetone, or mixtures of acetone with other organic solvents [23,24,25]. The clean-up of the extracts can be carried out with a number of techniques that differ significantly in simplicity and recovery of pesticides. Traditional liquid–liquid extraction (LLE) requires a huge amount of volatile solvents being threats to the environment and does not provide sufficient recovery. That is why the application of LLE for a large industrial-scale is not beneficial. Solid-phase extraction (SPE) is the next commonly used clean-up technique. Although SPE is simple and inexpensive, it is dedicated on a laboratory scale, moreover, its efficiency depends on the selection of the proper sorbent. Prousalis et al. [26] described a tedious but efficient analytical method for the simultaneous determination of carbendazim, thiabendazole, and o-phenylphenol residues in lemons involving a two-steps clean-up procedure with liquid–liquid partitioning after addition of an aqueous ammonia solution, and finally solid-phase extraction on polymeric reversed-phase. Fungicide recoveries from samples fortified at levels of 5 and 1 mg kg^−1^ were 81–85% for carbendazim, 96–98% for thiabendazole, and 81–106% for o-phenylphenol with coefficients of variation of 2.5–7.4%. In turn, more sophisticated techniques such as supercritical fluid extraction providing excellent results requires expensive equipment [27,28]. From this point of view, adsorption technology has the advantages of low treatment cost, the possibility of re-use, excellent efficiency, and can meet the requirements of industrial-scale. Activated carbon is the most commonly used adsorbent for this purpose. Recently, the preparation of Fe_3_O_4_ magnetic powder activated carbon (PAC) (Fe_3_O_4_-PAC) and MnFe_2_O_4_ magnetic PAC (MnFe_2_O_4_-PAC) were described for the in-depth removal of glyphosate pesticide from wastewater [29].

To summarize, there is still a dire need for elaboration on the effective and pro-environmental methods useful for the isolation of pigments from plants for the industry as well as their removal from extracts undergoing the aim for further analysis by sophisticated analytical instruments. In the presented work, the sorption capacity of selected plastics (polyvinyl chloride—PVC, polypropylene—PP, polyethylene—PE of different densities) granules was tested for the selective isolation of chlorophyll from spinach leaves extract. The comparison of the efficiency of the materials used for the isolation of chlorophyll gives an opportunity to create an effective and at the same time ecological method of obtaining this dye from plants. The proposed method is cheap, simple, efficient, avoiding the most hazardous toxic organic solvents. Furthermore, it proposes the new application for the most widely used plastics being products difficult for utilization., i.e., PVC, PE, and PP. The developed conditions make it possible to isolate or remove the pigment, thus satisfying the growing demand for pure natural compounds in the pharmaceutical and food industries, but can also be used to dye plastics with natural pigments. FTIR spectroscopy and molecular modeling were used to explain the sorption mechanism. In addition, the studies considered (i) sorption conditions, (ii) desorption conditions for chlorophyll and plastic reuse, (iii) the presence of selected fungicides used to protect plants against fungal infections as a potential contamination of the extracts, and the possibility of their co-sorption.

## 2. Experimental Section

### 2.1. Materials

HPLC organic solvents (acetone, acetonitrile, methanol, ethyl acetate) with reagent grade were obtained from Merck (Darmstadt, Germany). The sodium sulfate (Na_2_SO_4_) was obtained from P.O.Ch. (Gliwice, Poland). Water was deionized and purified by ULTRAPURE Milipore Direct-Q 3UV-R (Merck, Darmstadt, Germany). Reagent grade of chlorophyll *a* ~ 95% (HPLC, M = 893.49 g/mol) was obtained from Sigma-Aldrich (St. Louis, MO, USA). The following polymers were used: PVC (polyvinyl chloride (C_2_H_3_Cl)_n_), PP (polypropylene (C_3_H_6_)_n_), low-density polyethylene (C_2_H_4_)_n_, high-density polyethylene (C_2_H_4_)_n_. All materials in the form of granules: (PP) Polypropylene Moplen EP548R (Basell Orlen Polyolefins, Poland), with density 0.90 g/cm^3^, Melt Flow Rate 21 g/10 min (230 °C/2.16 kg), (LDPE) low-density Polyethylene Malen E FABS 23-D022 (Basell Orlen Polyolefins, Poland), with density 0.923 g/cm^3^, Melt Flow Rate 1,95 g/10 min (190 °C/2.16 kg), (HDPE) high-density Polyethylene Hostalen 4131B (Basell Orlen Polyolefins, Płock, Poland), density 0.941 g/cm^3^, Melt Flow Rate 2,2 g/10 min (190 °C/5.0 kg), (PVC-U) unplasticized poly(vinyl chloride) Alfavinyl GFM/4-31-TR (ALFA sp z o. o., Bielsko-Biała, Poland), with density 1.230 g/cm^3^. The materials used for the tests were in the form of granulate (3 mm diameter and 5 mm length) and in the form of a powder (with dimensions of 150 µm) obtained as a result of crushing the granules with mechanical mills.

Stock solutions (100 μg mL^–1^) of cyprodinil, chlorothalonil, and thiabendazone (Sigma-Aldrich, St. Louis, MO, USA) were prepared in acetone. Working standard solutions containing the analytes at various concentrations were prepared by diluting stock solutions, ensuring 2:1 the ratio of acetone:water. Samples of pesticide-free spinach extract were fortified by working solutions ensuring final pesticide concentration at the level of 1, 10, 50 µg mL^−1^.

### 2.2. Plant Extract

Fresh *Spinacia oleracea* L. mature leaves containing 8.53% dry matter were purchased from the local market. The extraction of the pigments from the biomass was described previously [30]. The cold acetone was used as the standard solvent for pigments extraction. The fresh spinach leaves (2.0 g) were cut into small pieces, triturated with cooled (4 °C) acetone (15 mL), and mixed with Na_2_SO_4_ (1.5 g). The mixture was macerating in a mortar for 10 min until the color of leaves disappeared. After centrifuging at 2500 rpm for 10 min, the resulting supernatant was directly injected into HPLC after appropriate dilution or used for further adsorption experiments.

### 2.3. HPLC Instrument

Chromatographic experiments were performed by the use a LaChrom HPLC Merck Hitachi (E.Merck, Darmstadt, Germany) model included a diode array detector (L-2455), column thermostat Jetstream 2 Plus (100375, Knauer), solvent degasser (L-7612), HSM software (Merck). The column was thermostated at 20 °C ± 0.1. The mobile phase was filtered through a Nylon 66 membrane filter (0.45 μm) Whatman (Maidstone, England) by the use of a filtration apparatus. The elution profile was monitored by a diode array detector (L-2455) (E.Merck, Darmstadt, Germany) by setting the wavelength according to the recorded spectrum in the range from 200 nm to 800 nm. Typical injection volumes were 20 μL, corresponding to the volume of the Rheodyne injector loop.

### 2.4. Quantitative Determination of Chlorophyll a

Stock solution was prepared by dissolving 1 mg of chlorophyll *a* in 10 mL of aceton:water (4:1 *v*/*v*). Solution was further diluted with mixture of aceton:water (4:1 *v*/*v*) to appropriate concentration. Reversed-phase chromatography conditions were detailed in Flieger et al. [30]. Briefly, pigments were eluted with acetonitrile/methanol/ethyl acetate (6:2:2, *v*/*v*) through Zorbax Eclipse XDB-C18 (150 mm × 4.6 mm I.D.) column with 5-μm (pore size: 80 Å, surface area: 180 m^2^/g) Agilent Technologies (Santa Clara, CA, USA) at a flow rate of 1.0 mL/min). The detection was carried out at 430 nm. The content of chlorophyll *a* was calculated by the external standard method. The calibration curve obtained by plotting the peak area against the nominal concentration was linear over a range of 0.5–100 mg L^−1^. The parameters of the linear regression function (*n* = 8, concentration in mg L^−1^) were: y = 364467(±17241)x − 585955(±709937), with R^2^ = 0.9890, s_e_ = 189588, F = 2446, where *R*^2^ is the correlation coefficient, *s*_e_ is the standard error of the regression, *F* is the value of the Fisher test of significance. The calibration curve were used for back-calculating chlorophyll-a concentration in the supernatant after the extraction procedure.

### 2.5. HPLC Conditions Used for the Pesticides Analysis

Pesticide concentrations were measured using HPLC with Zorbax Eclipse XDB-C18 column. The measurement conditions along with the chromatographic parameters are presented in Table 1. Recovery values were calculated as the ratio of the peak areas of the analytes from the fortified samples to the peak areas of standard solutions at appropriate concentration.

### 2.6. Batch Adsorption Experiments

Batch adsorption experiments were performed by a weighed amount of plastic (0.1–0.6 g). Adsorbent was added to a centrifugal tube containing 1 mL of extract prepared in acetone-water (*v*/*v*). The tubes were shaken and centrifuged at 9000× *g*. An aliquot of the supernatant was further analyzed by a HPLC procedure.

### 2.7. The Experimental Adsorption Isotherm

Adsorption experiments were performed by the weighed amounts of PVC (0.01–0.02 g). The each portion of adsorbent was added to the centrifugal tube containing 200 µL of the standard chlorophyll-a solution at concentration ranging from 0.5 to 100 mg L^−1^ (0.5; 0.75; 1.0; 2.5; 5.0; 7.5; 10.0; 25.0; 37.5; 50.0; 75.0; 100 mg L^−1^) prepared in acetone/water (4:1, *v*/*v*). The tubes were shaken at 20 °C for 1 h, and centrifuged at 9000× *g*. The solutions were removed by filtering through 0.45 μm syringe nylon-membrane filters, and the equilibrium chlorophyll-a concentrations in the filtrates were analyzed by an HPLC procedure described in Section 2.4. The isotherm of chlorophyll-a sorption on PVC was modeled by the best-fit offered by the Langmuir model of adsorption.

### 2.8. Molecular Modeling

The molecular modeling relied on the geometry optimization procedures carried out for porphyrin core of chlorophyll *a*, interacting with the CH_3_-CHCl-CH_3_ molecule that represents the fragment of a PVC chain. The main aim was to verify the hypothesis about the energetic favorability of the –Cl substituent interactions with Mg ion, present in the chlorophyll *a* core. The initial structure of the chlorophyll core was taken from the PDB database and refined within UFF force field [31] in the presence of interacting ligand (i.e., CH_3_-CHCl-CH_3_). The Avogadro 1.1.1 software was used for that purpose [32]. Several different initial orientations of the CH_3_-CHCl-CH_3_ molecule with respect to the chlorophyll core were tested; two of them, differing most significantly in geometries after UFF optimization, were selected for calculations at the higher level of theory. The final geometry optimization was performed within the DFT/B3LYP/6-311G** potential [33,34,35,36] either in vacuum or in the presence of implicit water (COSMO model [37]) by using the Gaussian09, Wallingford, UK, 2009 package [38]. An analogous procedure was repeated for unbound chlorophyll core and CH_3_-CHCl-CH_3_, and the final energies for all molecular systems were analyzed in terms of the CH_3_-CHCl-CH_3_-chlorophyll binding energies. This was done by subtracting the energy for the given complex from the sum of energies calculated separately for its substrates.

### 2.9. FT Raman Spectra

The Raman spectra of samples were obtained from a Raman Fourier transform spectrometer (Thermo Scientific, Waltham, MA, USA). Laser light at λ = 1064 nm was excited from Nd:YAG whose output power was 1 W. A Ge detector cooled with liquid nitrogen was used for detection. The spectra were recorded from 3700 to 150 cm^−1^, with a resolution of 8 cm^−1^. Each individual spectrum was the result of averaging 200 scans. The spectrum of each sample was obtained by averaging three individual scans of different PVC beads: pure or with chlorophyll. All spectral manipulation was carried out using Origin Pro 8.5 (OriginLab Corporation, Northampton, MA, USA). The spectra were baseline corrected (Omnic, Thermo Scientific, Waltham, MA, USA) and normalized to band at 2913 cm^−1^ (CH_2_ stretching vibration). The differential spectrum was also calculated by subtraction pure PVC spectrum from PVC with chlorophyll spectrum.

## 3. Results and Discussion

In the first part of the experiment, a solid–liquid extraction procedure to recover the chlorophylls from *Spinacia oleracea* L. was optimized. A screening of the most efficient plastic, the volume ratio of acetone-water, the mass of sorbent, and time of extraction were performed to optimize the extraction conditions. According to the so-called green solvents criteria, acetone belongs to recommended or at least preferred solvents [39,40]. Some studies indicate that acetone, which is a commonly used organic solvent, undergoes biodegradation [41]. Moreover, there is the possibility to produce acetone using biomass in a biotechnology-driven process from Green seaweed *Ulva lactuca* by *Clostridium acetobutylicum* and *Clostridium beijerinckii* [42].

### 3.1. Comparative Analysis of the Sorption Capacity of PVC, PE, PP towards the Isolation of Chlorophyll-a

**Figure 1:**
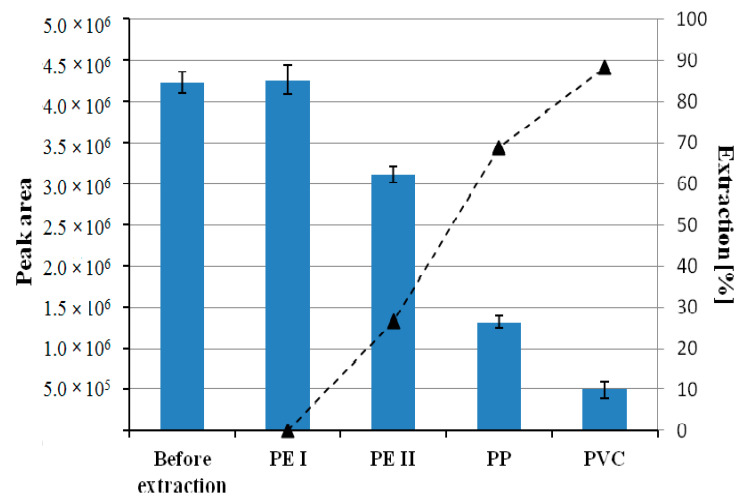
Peak area of chlorophyll *a* (the bar graph) and extraction efficiency [%] (the triangle signs) before extraction after mixing 0.5 mL of spinach leaf extract, 1.5 mL of acetone, and 1 mL of deionized water, with 0.3090 g of polyvinyl chloride (PVC), 0.3179 g of high-density polyethylene (HDPE), 0.3081 g of low-density polyethylene (LDPE), 0.3038 g of polypropylene (PP). The samples were placed in a rotor at maximum speed for 1 h and then determined by chromatography.

Four plastics were compared: Polyvinyl chloride (PVC), high-density polyethylene (HDPE), low-density polyethylene (LDPE), and polypropylene (PP) in terms of the sorption capacity towards chlorophyll *a*. The highest extraction efficiency was observed for polyvinyl chloride in comparison to other investigated plastics (Figure 1). Thus PVC has been chosen for subsequent experiments.

### 3.2. The Influence of the Volume Ratio of Acetone/Water (v/v) on Chlorophyll a Extraction (%) Efficiency

**Figure 2:**
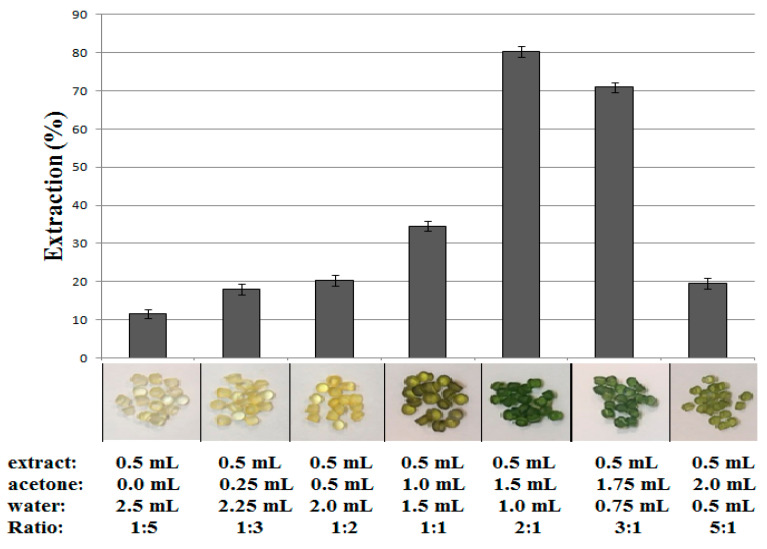
The influence of the volume ratio of acetone/water (ratio *v*/*v*) on chlorophyll *a* extraction efficiency (%) on 300 mg of PVC. The HPLC quantification was performed after 10 min at room temperature in batch experiments.

In order to evaluate the effect of adding water to acetone spinach leaf extract on the efficiency of chlorophyll *a* extraction by polyvinyl chloride, samples containing various ratios of acetone to water were tested. 0.3 g of polyvinyl chloride was added to each of the extract samples and placed in a rotator for 10 min at maximum speed. The content of chlorophyll *a* in the sample before extraction and after extraction was determined by HPLC in accordance with the method described in Section 2.3. The obtained results of the extraction efficiency, together with the composition of the analyzed samples and the photos of PVC after extraction, are shown in Figure 2. As shown in Figure 2, the best acetone to water ratio was 2:1. This proportion provides the highest percentage of chlorophyll *a* extraction by polyvinyl chloride in a batch experiment.

**Figure 3:**
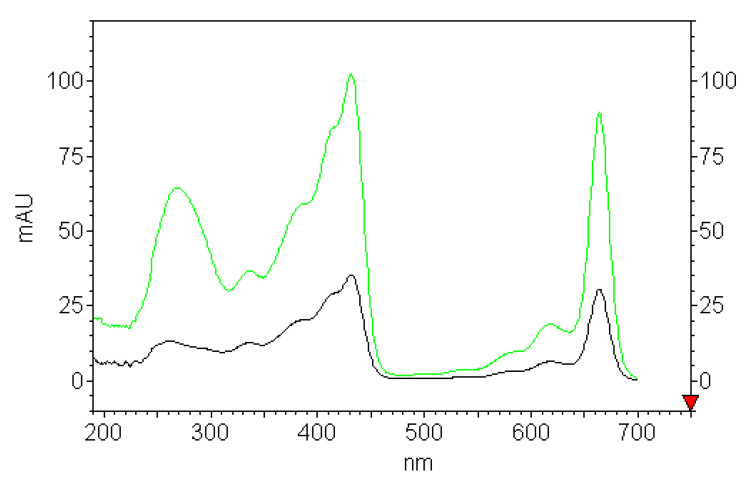
Absorption spectra of chlorophyll *a* in acetone-water (2:1) green line; and in acetone-water (1:5) black line.

The UV-Vis absorption spectra of above solutions were recorded after injection the samples into HPLC-DAD before plastics addition (Figure 3). In acetone as polar organic solvent with donor number 17.0; dielectric constant 20.7 F/cm; and dipolar moment 2.86 Debye, chlorophyll *a* is present in solution as a monomer solvated with the nucleophilic part of the solvent. The addition of water molecules representing the stronger ligand for chlorophyll *a* than acetone, with a donor number of 18.0, replaces the solvent ligands in key positions such as the Mg atom, 13C1dO carbonyl group, etc., allowing the formation of the chlorophyll *a* dihydrate and its photo-reactive dimer [43]. The pigment response in the water rich region up to 83% (the ratio of acetone to water, 1:5), where the solvent behaves almost as pure water, the aggregation processes are driven by hydrophobic interactions. The formation of spherical-shaped aggregates of chlorophyll *a* molecules in which the phytil chains are segregated in the inner part and the macrocyclic heads are exposed toward the bulk water solvent, forces moving the macrocycles toward the aqueous environment [44]. The presence of hydrophobic interactions in the water-rich solution, in the aim to minimize the exposure of the hydrophobic part of the molecule to water, is thermodynamically favorable and accounts for chlorophyll *a* aggregation. The observed changes present in the spectra of the investigated solutions containing acetone and different amount of water (83–25%) are related to the bulk dielectric constant and refractive index that only slightly influence the shape, but strongly affect λ max (Figure 3).

The aggregates of chlorophyll *a* molecules present in the water-rich solution with increasing water content up to 83.0% do not undergo adsorption on PVC, whereas the smaller addition of water about 33% (the ratio of acetone to water 2:1) created the best conditions for its adsorption with recovery up to 80% in one step.

### 3.3. The Influence of Time on the Sorption Efficiency of Chlorophyll a on Polyvinyl Chloride

**Figure 4:**
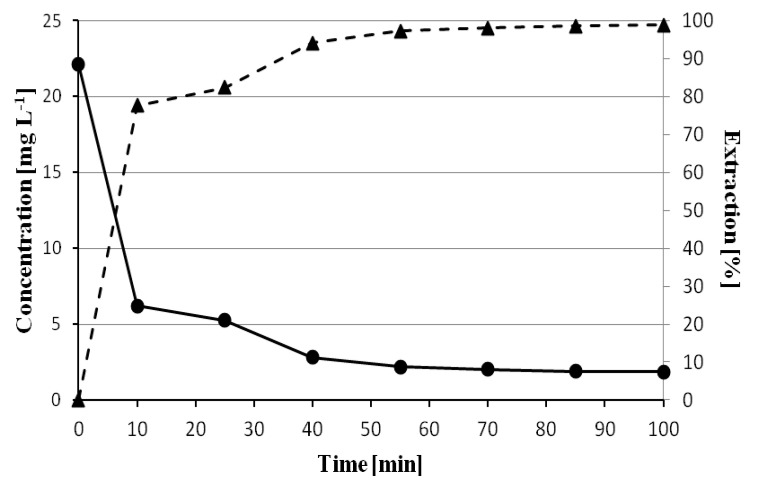
Optimization of the time needed to remove maximum chlorophyll *a* from solution containing 0.5 mL of spinach leaf extract, 1.5 mL of acetone, and 1 mL of deionized water by the use of 300 mg of polyvinyl chloride (PVC). The circles represent the decrease of the chlorophyll *a* concentration in time, the triangle signs show the relation between the percentage of its extraction vs. time.

In order to evaluate the effect of time on the sorption efficiency of chlorophyll *a* by polyvinyl chloride, samples were prepared containing: 0.5 mL of acetone extract from spinach leaves, 1.5 mL of acetone, and 1 mL of water. Then, 0.3 g of polyvinyl chloride was added. Samples were taken between 5 and 100 min and analyzed by chromatography assessing the percentage of chlorophyll *a* adsorbed. As can be seen in Figure 4, the maximum sorption value of 98.84% was obtained after 100 min of contact of the extract with polyvinyl chloride. A clear increase in the extraction yield can be seen in the first 55 min when the extraction percentage increases from 77.63% to 97.23%. In the next 30 min, the changes are not significant.

### 3.4. The Effect of Polyvinyl Chloride Mass on the Efficiency of Chlorophyll a Extraction

The flasks with a solution of 0.5 mL of spinach leaf extract, 1.5 mL of acetone, and 1 mL of deionized water were prepared. One of them was left as the reference sample, while the remaining were mixed with polyvinyl chloride (PVC) with different masses, and placed in the rotor with the highest speed for 1 h.

The results representing the percentage of chlorophyll-a removal by increasing masses of sorbent are collected in Table 2.

**Figure 5:**
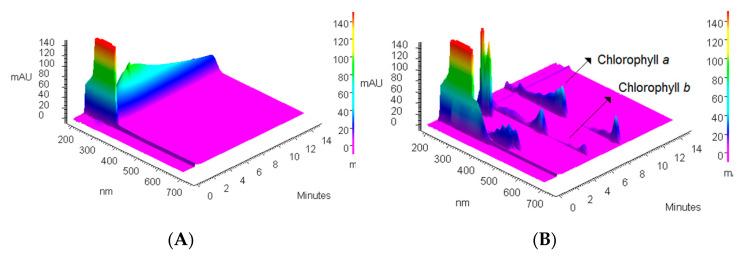

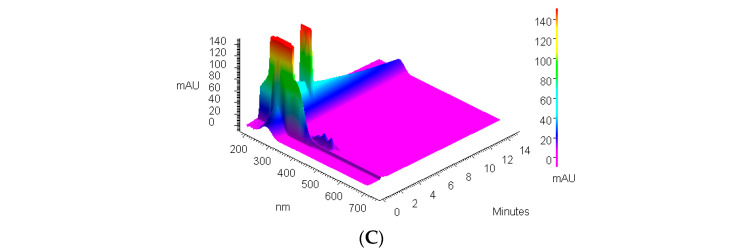
3D chromatograms of (**A**) 20 µL of blank (acetone-water 2:1 *v*/*v*), (**B**) 20 µL of spinach extract solution (0.5 mL, 5 mL acetone, 1 mL deionized water), (**C**) 20 µL of supernatant after PVC extraction (0.5 mL extract, 1.5 mL acetone, 1 mL water/0.60254 g PVC).

Considering the fact that 0.5 mL of extract of spinach leaves contains 30.125 µg of chlorophyll-a, 1.0351 g of PVC is able to remove almost all namely 98.92 ± 3.2% chlorophyll-a content through one extraction step. To illustrate the removal process, 3D chromatograms of blank showing spectrum of applied solvent (acetone-water) (Figure 5A), extract composition (Figure 5B), and one example from experiments collected in Table 2 proving the efficiency of the chlorophyll-a removal (Figure 5C), are presented in Figure 5.

### 3.5. The Experimental Adsorption Isotherm

**Figure 6:**
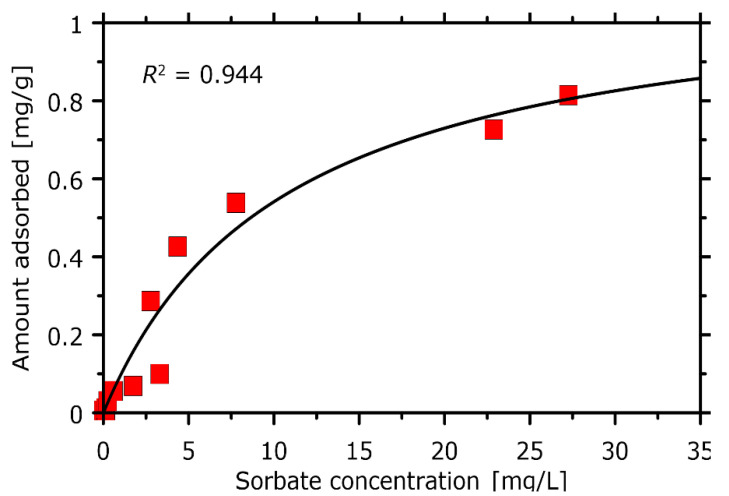
The experimental adsorption isotherm measured for the case of chlorophyll *a* sorption onto PVC. The technical details related to measurements are given in the text. The solid line represents the best-fit offered by the Langmuir model of adsorption. The estimated parameters of the Langmuir equation are equal to: 1.12 mg/g (adsorption capacity) and 0.0936 (Langmuir constant).

The experimental adsorption isotherm allows to interpret both the sorption capacity and the chlorophyll-PVC affinity in terms of the well-established models of adsorption. Figure 6 shows the graphical illustration of the collected data and the prediction of the Langmuir model of adsorption with the parameter values adjusted to provide the maximal value of the determination coefficient (*R*^2^). The good applicability of the Langmuir model (*R*^2^ = 0.944) suggest the low scatter of sorbent-sorbate affinities across the binding sites and the uniform mechanism of sorption. This is in line with the postulated mechanism of binding that relies on (uniform) coordination of the –Cl group by the magnesium ion present in the chlorophyll structure. The estimated sorption capacity is equal to 1.12 mg/g which is moderate value in the context of sorption capacities characteristic for other solid sorbents that have been used to remove chlorophyll from solution in the past [45,46,47,48,49]. The exceptionally high sorption capacity is characteristic of micro- and mesoporous sorbents such as activated carbons (~20 mg/g) whereas remaining sorbents exhibit a very broad range of sorption capacities (varying from 0.3 to 10 mg/g). Due to the fact of highly diverse experimental conditions (different temperatures, solvents, etc.), it is hard to perform a more quantitative analysis of the sorption effectiveness in these systems. It is also worth noting that most of the already tested sorbents are modified inorganic minerals (e.g., sepiolite, bentonite, mesoporous silica), contrary to the presently considered PVC.

### 3.6. Desorption Studies

**Figure 7:**
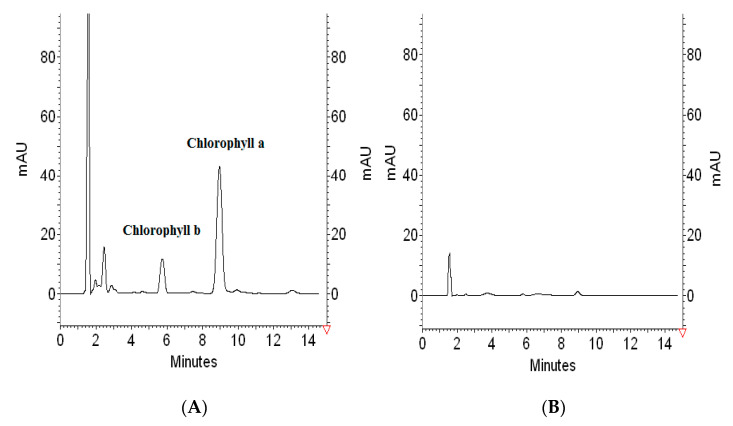
Chromatograms of the supernatants obtained from PVC (0.6 g) treated by 0.5 mL extract +1.5 mL acetone +1 mL water Figure 1 h, after elution by the use of 3 mL acetone (**A**), or methanol (**B**).

Figure 7 presents the results of the back-extraction of chlorophyll *a* achieved by using regenerating solutions containing pure methanol or acetone. The experiments were conducted at 20 °C. The results have shown that chlorophyll *a* was re-extracted to acetone by the volume of 3 mL at room temperature. The extraction recovery has achieved 91% at one step. The back-extraction of chlorophyll *a* was suppressed to 2%, when the pure acetone was replaced by pure methanol, and to 21% when acetonitrile was used as eluting solvent. It has been proven that by the stripping of the PVC by a regenerating solution containing pure acetone, satisfactory extraction efficiency has been achieved, but also enabled to regenerate PVC sorbent.

### 3.7. Pesticides Contamination

As fungicides, such as cyprodinil, chlorothalonil, and thiabendazone, belonging to planar pesticides are commonly used on high chlorophyll commodities, they were spiked into the spinach extract at three concentration levels ranging between 1–50 μg/mL of sample. Spiked samples were extracted using PVC granules. For pesticide quantification, high-performance liquid chromatography (HPLC) was used with UV detection at their analytical wavelength chosen on the basis of recorded spectra in the range of 200–400 nm. Results are presented in Table 3 showing the % of pesticide residues in the extract. It appeared that PVC is very effective in removing not only chlorophyll, but also cyprodinil and chlorothalonil. Significant losses of these pesticides were observed at each concentration level: For cyprodinil in the range of 59.95–63.35%, and for chlorothalonil in the range of 42.54–75.49%. In contrast, PVC applied as a sorbent was able to remove chlorophyll with little or no loss of thiabendazone. This experiment shows the possibility to remove the most common pesticides, namely cyprodinil and chlorothalonil from plant extract by PVC. On the other hand, effective isolation of pure chlorophylls is possible if the initial plant is free of them. Thiabendazone is the only pesticide which can be allowed in the cleanup protocol of the green pigments. The repeatability method was estimated by three independent analyses of fortified samples. The concentration-dependent RSD% values range from 0.7 to 15.03%, demonstrating a satisfactory repeatability.

### 3.8. Sorption Mechanism

**Figure 8:**
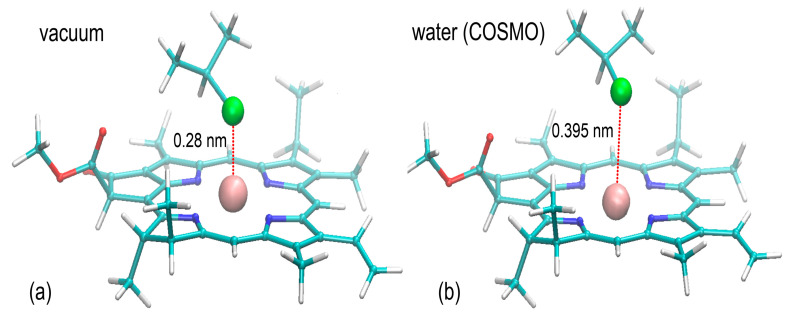
The structures of the CH_3_-CHCl-CH_3_ molecule interacting with the core of chlorophyll *a*. The structures were obtained in the result of ab initio geometry optimization carried out at the DFT/B3LYP/6-311G** level of theory either in the absence (**a**) of presence (**b**) of implicit water solvent. The Mg^2+^ cation and the coordinating Cl atom are shown as balls.

The initially accepted mechanism of binding relied on the assumption about coordination of the Mg^2+^ cation by the –Cl substituent present in the PVC chain. This pattern of interactions, displayed in the UFF-optimized structures appeared to be characteristic also of the final structures, representing the minima on the DFT potential energy surface calculated for vacuum simulations. The representative structure is shown in Figure 8. The strong interactions between Mg and Cl are confirmed by the corresponding interatomic distances varying between 0.280 and 0.283 nm, which agree with the Mg-Cl bond length in ‘crowded’ molecular complexes [50]. Upon the presence of implicit solvent, those distances increase to 0.395–0.413 nm, indicating loosening of the Mg-Cl interactions. Interestingly, the corresponding distances between porphyrin rings and the central aliphatic carbon of CH_3_-CHCl-CH_3_ vary only slightly, increasing from ~0.37 to ~0.395 (see Figure 8).

The interatomic distances discussed above are reflected by the calculated binding energies corresponding to the environment of the given system. For the vacuum systems the determined binding energies are more favorable and equal to 18.8–25.5 kJ/mol, depending on the system. In the presence of water, those values are reduced to 9.3–11.9 kJ/mol. Still, even those smaller values correspond to a highly favorable binding and show the existence of attractive interactions between PVC and chlorophyll.

The observed differences can be interpreted in terms of the competitive interactions of electronegative ligands for the Mg^2+^ ion. In the absence of water, the interactions between Cl and Mg are much more attractive and result in more favorable binding, whereas the binding strength is largely reduced upon introducing the implicit water. The oxygen atoms of water are capable to compete for interactions with Mg^2+^, collectively diminishing the strength of the Cl-Mg^2+^ attraction. Thus, it is expected that in the presence of water, PVC-chlorophyll binding is driven not only by the Cl-Mg^2+^ coordination, but also by interactions of the CH-π type between the porphyrin rings and aliphatic hydrogens of PVC. However, due to both large size of involved molecules and extremely high conformational flexibility of the PVC chain, this problem can hardly be accurately addressed by the ab initio calculations. Nevertheless, the presented calculations show that PVC exhibits favorable, attractive interactions toward chlorophyll of magnitude sufficient to create stable molecular complexes.

### 3.9. FT-Raman Spectroscopy

**Figure 9:**
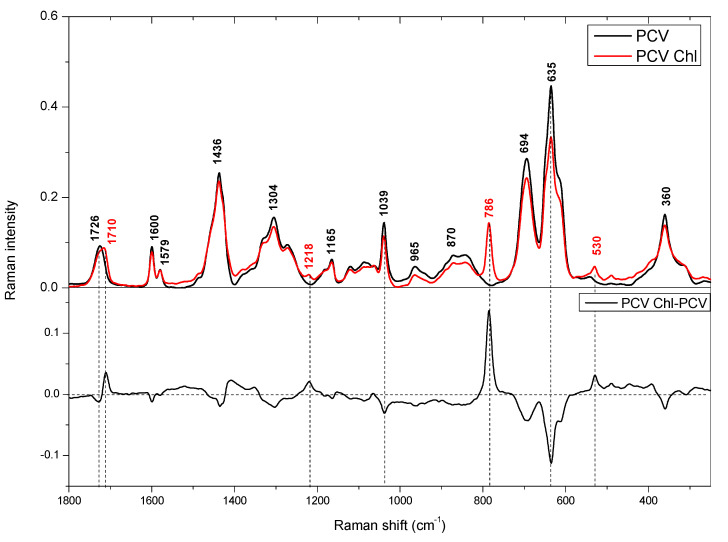
The FT Raman spectra of PVC (black line) and PVC with adsorbed chlorophyll (PVC-Chl, red line) in the range 1800–250 cm^−1^ (upward panel). Downward panel presents differential spectrum.

The FT Raman spectrum of pure PVC is characterized by intense bands at 635 and 694 cm^−1^ assigned to stretching vibration of C-Cl (Figure 9) [51]. However, bands at 1039, 1579, 1600, and 1726 cm^−1^ clearly denote a phthalate additive to PVC [52].

The Raman spectrum of PVC with chlorophyll showed a slight shift of band at 1726 to 1710 cm^−1^. In PVC this band is probably connected with C=O stretching vibration in phthalate additive and its shift after adsorption of chlorophyll can be proof of interaction between PVC and chlorophyll via C=O group [53]. On the other hand, intensity of bands at 635 and 694 cm^−1^ (stretching of C-Cl) decreased after adsorption of chlorophyll (negative bands in differential spectrum) could be proof of interaction between Cl with Mg present in chlorophyll. Additionally, in spectrum of PVC with chlorophyll the strong band at 786 cm^−1^ appeared. This band is not present in spectrum of pure chlorophyll in acetone (there can be found band at 801 cm^−1^) [54]. The shift of this band toward lower frequencies could be proof of interaction of PVC with chlorophyll via central magnesium atom. Additionally, the weak bands at 1210 and 530 cm^−1^ appeared, however the nature of those bands was not determined.

## 4. Conclusions

Chlorophylls are used in a wide range of applications in medicine, pharmacy, cosmetics, and the food industry. To replace the organic solvents which are commonly applied for their extraction from biomass, several plastics (PVC, PP, PE) were screened on their extraction ability of chlorophylls *a* and *b* from spinach leaves. The best recovery was obtained for PVC. The molecular modeling, as well as FT Raman spectra, confirm that PVC containing electronegative chlorine exhibits favorable, attractive interactions toward chlorophyll of a magnitude sufficient to create stable molecular complexes. It should be pointed out that the Raman spectra definitely prove the existence of the interactions between PVC and chlorophyll, displayed by the band at 786 cm^−1^. The chemical nature of the considered compounds and the results of the complementary DFT calculations allow to associate this band with the formation of the Cl-Mg bond.

The optimized operational extraction conditions (mass of sorbent, water-acetone ratio, and time), enabled a maximum extraction yield of chlorophylls of 98%. After the extraction step, the chlorophyll-rich sorbent was re-extracted, leading to the recovery of 91% of chlorophylls in acetone in one step, adding the possibility to its simple re-use. The obtained results show the potential of PVC to extract highly hydrophobic compounds from biomass, without requiring an additional recovery or purification step. The estimated sorption capacity for PVC is equal to 1.12 mg/g which is a moderate value in the context of sorption capacities. However, it should be emphasized the exceptional ease of use of PVC grains, which does not even require centrifugation in batch experiments. Noteworthy is also the possibility of using the demonstrated sorption of green dyes by PVC for its coloring. This aspect of potential industrial applications requires further research to determine the optimal conditions, such as grain size. This direction is interesting, taking into account the fact that chlorophyll is a natural, ecological pigment and it is stabilized due to interactions with polymers [55].

Furthermore, PVC is very effective in removing not only chlorophyll, but also cyprodinil, chlorothalonil. In turn, thiabendazone is the only pesticide that does not disturb the cleanup process of the green pigments. Performed experiments prove that PVC can be easily applied as an environmentally safe sorbent to remove chlorophylls from extracts as well as obtaining them in clean-state on an industrial scale if proper fungicide was applied during the production step.

## Figures and Tables

**Table 1:** The HPLC conditions with the chromatographic parameters obtained for the pesticides.

Pesticide log *P*	Mobile Phase ACN/Water	t_R_ (min)	λ (nm)	As	N
Thiabendazole 2.47 *	30% (*v*/*v*)	3.59	301	0.89	28,667
Cyprodinil 4.0 **	60% (*v*/*v*)	8.09	205	0.97	55,313
Chlorothalonil 3.05 ***	60% (*v*/*v*)	7.08	230	1.02	63,540

* https://pubchem.ncbi.nlm.nih.gov/compound/Thiabendazole (accessed on 25 March 2005); ** https://pubchem.ncbi.nlm.nih.gov/compound/Cyprodinil (accessed on 24 June 2005); *** https://pubchem.ncbi.nlm.nih.gov/compound/Chlorothalonil (accessed on 27 March 2005).

**Table 2:** The removal of chlorophyll-a from a solution containing 0.5 mL of spinach leaf extract, 1.5 mL of acetone, and 1 mL of deionized water by the use of different masses of PVC sorbent in the batch adsorption experiments.

PVC (g)	Peak Area	Amount of Chlorophyll-a Sorbed (µg)	Removal (%)	SD%
0.3037	387,749	26.34	87.38	3.8
0.3988	137,536	28.79	95.52	2.4
0.5092	69,884	29.46	97.73	2.6
0.6025	47,132	29.68	98.47	4.0
1.0351	33,184	29.79	98.92	3.2

**Table 3:** Average recoveries of investigated pesticides and %RSD values.

Pesticide	Fortification Level of Spinach Extract (µg mL^−1^)	Average Recovery ±SD, *n* = 3	Repeatability RSD (%)
Thiabendazole	50	98.70 ± 0.71	0.72
	10	93.32 ± 1.30	1.39
	1	100.39 ± 3.91	3.89
Cyprodinil	50	26.65 ± 2.53	9.48
	10	41.05 ± 1.52	3.70
	1	34.89 ± 5.24	15.03
Chlorothalonil	50	24.51 ± 0.62	2.55
	10	57.46 ± 1.75	3.04
	1	29.39 ± 1.06	3.60

## Data Availability

The reported results can be found in the Department of Analytical Chemistry of Medical University of Lublin.
